# Intense Sweetness Surpasses Cocaine Reward

**DOI:** 10.1371/journal.pone.0000698

**Published:** 2007-08-01

**Authors:** Magalie Lenoir, Fuschia Serre, Lauriane Cantin, Serge H. Ahmed

**Affiliations:** University Bordeaux 2, Université Bordeaux 1, CNRS, UMR 5227, Bordeaux, France; James Cook University, Australia

## Abstract

**Background:**

Refined sugars (e.g., sucrose, fructose) were absent in the diet of most people until very recently in human history. Today overconsumption of diets rich in sugars contributes together with other factors to drive the current obesity epidemic. Overconsumption of sugar-dense foods or beverages is initially motivated by the pleasure of sweet taste and is often compared to drug addiction. Though there are many biological commonalities between sweetened diets and drugs of abuse, the addictive potential of the former relative to the latter is currently unknown.

**Methodology/Principal findings:**

Here we report that when rats were allowed to choose mutually-exclusively between water sweetened with saccharin–an intense calorie-free sweetener–and intravenous cocaine–a highly addictive and harmful substance–the large majority of animals (94%) preferred the sweet taste of saccharin. The preference for saccharin was not attributable to its unnatural ability to induce sweetness without calories because the same preference was also observed with sucrose, a natural sugar. Finally, the preference for saccharin was not surmountable by increasing doses of cocaine and was observed despite either cocaine intoxication, sensitization or intake escalation–the latter being a hallmark of drug addiction.

**Conclusions:**

Our findings clearly demonstrate that intense sweetness can surpass cocaine reward, even in drug-sensitized and -addicted individuals. We speculate that the addictive potential of intense sweetness results from an inborn hypersensitivity to sweet tastants. In most mammals, including rats and humans, sweet receptors evolved in ancestral environments poor in sugars and are thus not adapted to high concentrations of sweet tastants. The supranormal stimulation of these receptors by sugar-rich diets, such as those now widely available in modern societies, would generate a supranormal reward signal in the brain, with the potential to override self-control mechanisms and thus to lead to addiction.

## Introduction

Sweet taste perception is an innate capacity that depends on two G-protein-coupled subunit receptors, T1R2 and T1R3, located on the tongue [1], [2]. The stimulation of these receptors by diets rich in sweet tastants, such as, for instance, sugar-sweetened beverages (soft drinks, colas, fruit beverages), generates a sensation that most humans and other mammals, including rodents, find intensely rewarding [3]–[6]. Once reserved to a small elite, the consumption of highly sweetened diets is now highly prevalent in developed countries and is escalating elsewhere [7], [8]. Though difficult to estimate, sweet sensations evoked by sugar-sweetened foods and drinks are probably one of the most precocious, frequent and intense sensory pleasures of modern humans [7], [9]. However, the current pursuit of sweet sensations far exceeds metabolic needs and is thought to contribute, together with several other factors [10]–[13], to drive the current obesity epidemic [7], [14].

The passive overconsumption of sugar-sweetened diets has often been compared to drug addiction, though this parallel was based until very recently more on anecdotal evidence than on solid scientific grounds. More recently, mounting evidence from experimental research on animals, especially rats, have unearthed deep commonalities between overconsumption of sugars and drug addiction [15]–[17]. First, both sweet tastants [18], [19] and drugs of abuse [20], [21] stimulate dopamine signaling in the ventral striatum, a brain signaling pathway critically involved in reward processing and learning [22], [23]. Second, both cross-tolerance [24], [25] and cross-dependence [26]–[28] have been observed between sugars and drugs of abuse. For instance, animals with a long history of sucrose consumption become tolerant to the analgesic effects of morphine [25]. In addition, naloxone–an opiate antagonist–precipitates in rats with sugar overconsumption some of the behavioral and neurochemical signs of opiate withdrawal [28]. This latter observation is important because it shows that overconsumption of sugar-sweetened beverages may induce a dependence-like state. Finally, recent neuroimaging studies in humans have recently discovered neuroadaptations in the brain of obese individuals that mimic those previously observed in individuals addicted to cocaine and other drugs of abuse [29], [30].

Overall, there are many behavioral and biological commonalities between sugar-sweetened beverages and drugs of abuse. However, the addictive potential of the former relative to the latter is much less clear. Previous research showed that concurrent access to highly sweetened water (saccharin plus glucose) can reduce self-administration of low doses of cocaine in non-dependent rats [31], [32], suggesting that sweetened water may surpass cocaine reward–one of the most addictive and harmful substance currently known [33]. Whether this effect results from a genuine preference for intense sweetness or other factors (e.g., use of a suboptimal dose of cocaine and/or lack of cocaine dependence) has not been established yet, however. The present series of experiments was designed to directly address this question. We developed a discrete-trials choice procedure to measure the reward value of an intense sweet taste relative to intravenous cocaine. This procedure was first tested in non-restricted, naïve rats to determine how, without any prior experience with cocaine or intense sweetness, animals learn to differentially value both types of reward. Then, the same procedure was applied to rats following an extended access to cocaine self-administration. Previous research showed that with prolonged access to cocaine, most rats develop the major signs of addiction, including drug intake escalation [34], compromised brain reward processing [35] and difficulty to stop drug seeking despite negative consequences [36].

## Results

**Figure 1:**
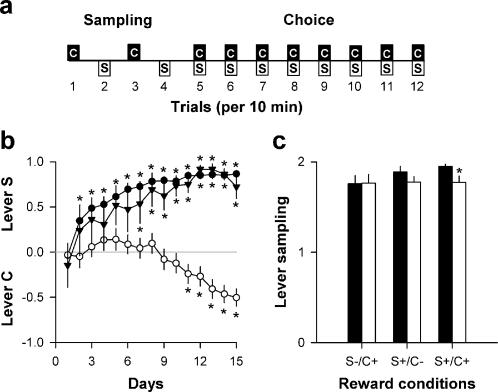
Choice between saccharin and cocaine. a, Schematic representation of the choice procedure. Each choice session was constituted of 12 discrete trials, spaced by 10 min, and divided into two successive phases, sampling (4 trials) followed by choice (8 trials). S, saccharin-associated lever; C, cocaine-associated lever. b, Choice between levers C and S (mean±SEM) across reward conditions and as a function of time (open circle: S-/C+ condition; closed triangle: S+/C- condition; closed circle: S+/C+ condition). The horizontal gray line at 0 indicates the indifference level. Values above 0 indicate a preference for lever S while values below 0 indicate a preference for lever C. *, different from the indifference level (*P*<0.05, *t*-test). c, Sampling (mean±SEM of the last 3 days) of lever S (black bars) and lever C (white bars) across reward conditions. *, different from lever S (*P*<0.05, Fisher's LSD test after a two-way analysis of variance).

Drug-naïve rats with no prior experience with refined sugar or artificial sweetener were allowed to choose 8 times per day between two mutually exclusive levers (Fig. 1a): responding on one lever (lever C) was rewarded by a behaviorally effective dose of cocaine (0.25 mg, i.v.) while responding on the other lever (lever S) was rewarded by a 20-s access to water sweetened with saccharin (0.2%) (see Materials and Methods). Importantly, each day before making their choices, rats were allowed to alternatively sample each lever 2 times to learn their respective reward value (Fig. 1a). Different groups of animals were tested under 3 reward conditions. Under the S-/C+ condition (*N* = 30), only responding on lever C was rewarded (+) by cocaine delivery; responding on lever S was not rewarded (-). Under the S+/C- condition (*N* = 9), only responding on lever S was rewarded by saccharin access; responding on lever C was not rewarded. Finally, under the S+/C+ condition (*N* = 43), both levers were rewarded by their corresponding rewards. There was more rats in the S-/C+ or S+/C+ condition than in the S+/C- condition because more experiments were conducted in these former conditions to assess the determinants of choice between saccharin and cocaine (dose, delay, effort, reversal, calorie input, thirst).

On day 1 and whatever the reward conditions, rats were indifferent to both levers, showing that there was no preexisting bias or preference in our setting. As expected, however, with repeated testing, reward conditions considerably influenced the evolution of lever choice [Condition×Day: *F*(28,1106) = 8.71, *P*<0.01] (Fig.1b). Under the S-/C+ condition, rats displayed no preference until day 9, when they shifted toward preferring lever C. This preference became statistically reliable on day 11. Similarly, under the S+/C- condition, rats rapidly acquired a preference for lever S which became statistically reliable on day 7. More surprisingly, under the S+/C+ condition, rats immediately developed a strong and stable preference for lever S which became statistically significant on day 2. This preference was indistinguishable from that exhibited by rats in the S+/C- condition [*F*(14,700) = 0.41, NS] (Fig. 1b). In addition, after stabilization of behavior, the latency to select lever S in the S+/C+ condition (14.5±5.0 s, means±SEM of the last 3 stable days) was similar to that in the S+/C- condition (6.5±2.4 s) [*t*(50)<1], showing that rats chose saccharin over cocaine without hesitation, as if lever C was not rewarded by cocaine.

The strong preference for saccharin under the S+/C+ condition was not due to a failure to learn the value of lever C. Indeed, from day 7 onward, rats sampled lever C almost maximally, though slightly less than lever S, before being allowed to make their choices (Fig. 1c). Thus, despite near maximal cocaine sampling, rats under the S+/C+ condition acquired a preference for lever S as quickly as rats under the S+/C- condition. This finding also shows that cocaine had no positive or negative influence on saccharin acceptance and/or preference in the present choice setting. Finally, after stabilization of behavior, the latency to sample lever C (48.5±10.2 s, means±SEM of the last 3 stable days) was significantly greater than the latency to sample lever S (5.6±1.7 s) [*F*(1,42) = 17.44, *P*<0.01]. This difference shows that animals have effectively learned that each lever is associated with a different outcome.

**Figure 2:**
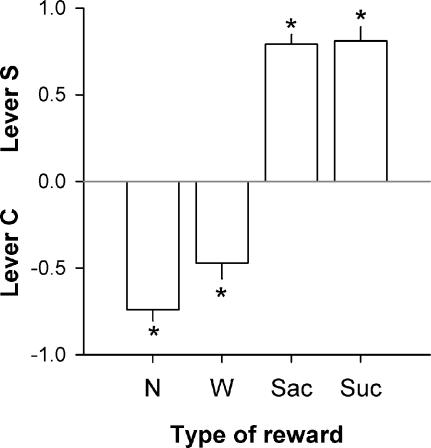
Choice between lever C and no fluid (N), water (W), saccharin (Sac, 0.2%) or sucrose (Suc, 4%). The horizontal gray line at 0 indicates the indifference level. Values above 0 indicate a preference for lever S while values below 0 indicate a preference for lever C. *, different from the indifference level (*P*<0.05, *t*-test). Each reward type (N, W, Sac and Suc) was tested at least 5 times in a row until stabilization of behavior. Bars represent the means (±SEM) of the last 3 stable days. The first 3 reward conditions (N, W and Sac in this order) were tested in the same group of animals (*N* = 10) while the sucrose condition was tested in a separate group (*N* = 10).

It is important to note that the preference for saccharin was not attributable to thirst or drinking behavior *per se* because rats preferred cocaine over mere water (Fig.2). Finally, the preference for saccharin was not due to its unnatural ability to induce sweetness without calories because the same preference was also observed with an equipotent concentration of sucrose (4%) (Fig.2).

**Figure 3:**
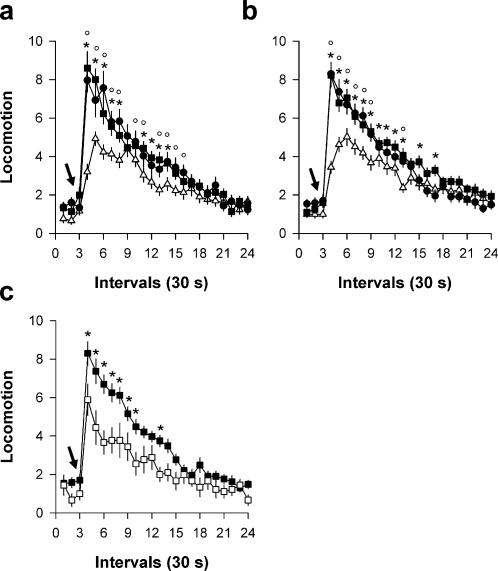
Cocaine-induced locomotion in rats tested under. a, the S-/C+ condition, or b, the S+/C+ condition. Locomotion (i.e., mean number of cage crossings±SEM) was measured during 10 min after the first cocaine self-injection (0.25 mg, i.v.) of the day (open triangle: day 1; closed circle: day 5; closed square: day 15). *, day 5 different from day 1; °, day 15 different from day 1 (*P*<0.05, Fisher's LSD test). c, Effects of the first cocaine self-injection in rats initially trained under the S+/C- condition and tested for the first time under the S+/C+ condition on day 16. These effects (open square) were compared to the effects of cocaine on day 15 in rats initially trained under the S+/C+ (closed square). *, *P*<0.05, Fisher's LSD test. The arrow in all graphs indicates the intravenous injection of cocaine.

To directly assess the behavioral efficacy of cocaine in the discrete-trials choice procedure, we measured the ability of the first cocaine self-injection of the day to induce locomotion on day 1, 5 and 15. As expected, in rats which acquired a preference for lever C under the S-/C+ condition, cocaine induced a rapid increase in locomotion which peaked 1 min post-injection and then returned gradually to baseline within the 10-min inter-trial interval (Fig. 3a). This psychomotor effect increased even further after repeated cocaine exposure [Day×Intervals: *F*(40,1160) = 5.06, *P*<0.01], a well-established phenomenon, called behavioral sensitization. Sensitization to cocaine was maximal as soon as day 5 and remained stable until the end of the experiment, despite additional cocaine exposure (Fig. 3a). Importantly, a behavioral sensitization of a similar magnitude was also observed in rats which acquired a strong preference for lever S under the S+/C+ condition [Day×Intervals: *F*(40,1680) = 6.57, *P*<0.01] (Fig. 3b). To test the specific contribution of saccharin consumption to the induction of sensitization in the S+/C+ condition, rats initially tested under the S+/C- condition were tested under the S+/C+ condition on day 16. These rats were much less sensitive to cocaine than rats initially trained under the S+/C+ condition [Group×Intervals: *F* (20, 1000) = 1.66, *P*<0.05] (Fig. 3c). This observation clearly shows that saccharin consumption *per se* has little impact on sensitization under the S+/C+ condition and thus that the very few doses of cocaine consumed in the S+/C+ condition (mostly during sampling) were sufficient in themselves to induce sensitized responding. Thus, rats preferred saccharin over cocaine despite being fully responsive and sensitized to (and by) cocaine.

**Figure 4:**
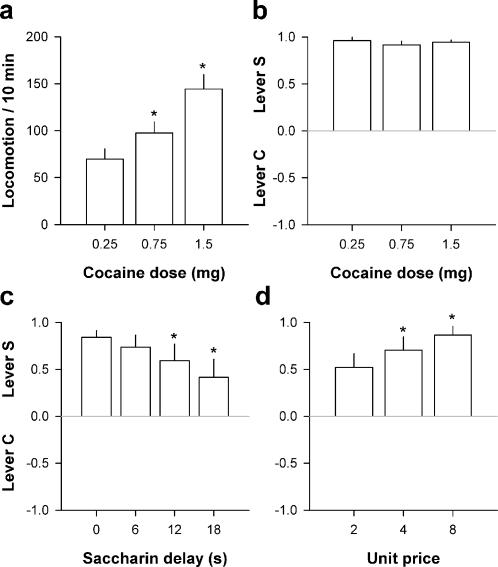
Pharmacological and economic determinants of cocaine choice. a, Cocaine-induced locomotion as a function of dose. Locomotion (i.e., mean number of cage crossings±SEM) was measured during 10 min after the first cocaine self-injection of the first day of each dose substitution. b, Choice between levers C and S (mean±SEM) as a function of dose. c, Choice between levers C and S (mean±SEM) as a function of delay between response and saccharin delivery. *, different from the shortest delay (*P*<0.05, Fisher's LSD test after one-way ANOVA). d, Choice between levers C and S (mean±SEM) as a function of reward price. *, different from the lowest price (*P*<0.05, Fisher's LSD test after one-way ANOVA). The values of each variable (dose, delay and price) were tested at least 5 times in a row until stabilization of behavior. Bars represent the means of the last 3 stable days.

It is possible that though efficacious in inducing locomotion and sensitization, the dose of cocaine was nevertheless too low to surpass the rewarding effects of saccharin. To address this question, a subgroup of rats (*N* = 11) trained under the S+/C+ condition was tested with increasing i.v. doses of cocaine (0.25-1.5 mg). The highest dose was near but lower than the convulsive dose (i.e., 3 mg) in our conditions. As expected, increasing the dose of cocaine induced a dose-dependent increase in locomotion, as measured during 10 min after the first cocaine self-injection of the first day of each dose substitution [*F*(2,20) = 18.77, *P*<0.01] (Fig. 4a). However, regardless of the dose available, rats continued to prefer lever S over lever C [*F*(2,20) = 0.07, NS] (Fig. 4b). Thus, rats preferred saccharin despite a near maximal level of cocaine stimulation. Though the intravenous route of administration allows for rapid and intense drug effects–which explains why this route is often selected by heavy drug users–there is still a brief, incompressible delay between lever pressing and onset of cocaine effects. This delay of action was estimated at 6.2±0.2 s in the present study (see Materials and Methods). Similarly, the neurochemical effects of cocaine peak between 4 and 20 s after the onset of an intravenous injection [37]. In contrast, the delay between response and onset of saccharin drinking was less than 2 s. This difference of delay, though small, could nevertheless explain the preference for saccharin whose rewarding effects are more immediate than those of cocaine. To test the contribution of this factor, saccharin delivery was systematically delayed after selection of lever S (0–18 s) in a subgroup of rats (*N* = 11) while the delay of cocaine delivery remained constant. Increasing the delay of saccharin delivery induced a slight decrease in selection of lever S [*F*(3,30) = 6.58, *P*<0.01] (Fig. 4c). This increase was not sufficient, however, to reverse the preference for lever S in favor of lever C. Thus, rats preferred saccharin even when its delay was equal to or above the delay of cocaine effects. Finally, we assessed in another subgroup of rats (*N* = 10) the effects of the reward price (i.e., the number of lever presses required to obtain a reward) on choice. In some cases, increasing reward price can induce a shift in preference [38]. However, increasing reward price from 2 to 8 responses/reward did not reverse but instead increased the preference for lever S [*F*(2,18) = 8.04, *P*<0.01] (Fig. 4d). Thus, regardless of the price, rats preferred saccharin over cocaine.

**Figure 5:**
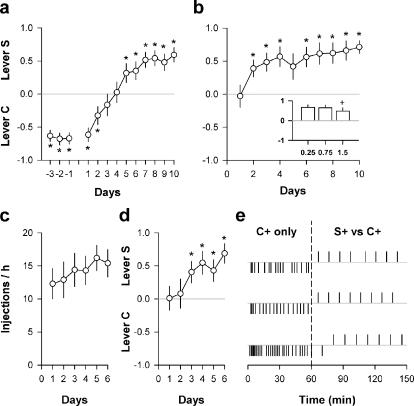
Choice between saccharin and cocaine as a function of drug history. a, Reversal of preference in rats which had acquired a preference for lever C under the S-/C+ condition. The first 3 days (-3 to -1) correspond to baseline choice under the S-/C+ condition. The next 10 days correspond to choice after the shift to the S+/C+ condition. b, Choice between levers C and S (mean±SEM) after cocaine intake escalation. Insert: Choice between levers C and S as a function of the dose. c, Cocaine self-injections (mean±SEM) during the hour preceding choice in the modified discrete-trials choice procedure. d, Choice between levers C and S (mean±SEM) during cocaine intoxication. e, Representative individual distributions of cocaine rewards (downward ticks) or saccharin rewards (upward ticks) within the last testing session. The vertical dashed line separates the 1-hour exclusive access to cocaine self-administration (C+ only) from the subsequent 8 discrete choices (S+/C+ condition). *, different from the indifference level (*P*<0.05, *t*-test); +, different from the lowest dose (*P*<0.05, Fisher's LSD test after one-way ANOVA).

The previous series of experiments involved initially drug-naïve individuals with no prior history of cocaine self-administration. To determine whether drug history influences the choice between saccharin and cocaine, a subgroup of rats (*N* = 24) which had acquired a stable preference for lever C under the S-/C+ condition were subsequently tested under the S+/C+ during 10 days. Despite an initial, stable preference for lever C, rats rapidly reversed their preference in favor of lever S when both levers were rewarded (Fig. 5a). The proportion of rats that preferred lever C (i.e., mean selection of lever C of the last 3 days>60%) after preference reversal did not differ significantly from that recorded in initially drug-naïve rats (8.3 versus 2.3%, *z*<1.96). In addition, the preference for saccharin developed even in rats (*N* = 11) with a long history of cocaine self-administration (6 h per day, during 3 weeks). In the present study, despite 3 weeks of extended access to cocaine self-administration and a large escalation of cocaine consumption [from 7.34±2.50 to 26.04±1.21 mg/day; *F*(16,160) = 15.98, *P*<0.01], rats rapidly acquired a strong and stable preference for lever S over lever C (Fig. 5b). The proportion of rats with prolonged access to cocaine that preferred lever C after 10 days of choice did not differ from that recorded in initially drug-naïve rats (0.0 versus 2.3%, *z*<1.96). Despite a small decrease in selection of lever S at the highest dose, the preference for lever S in rats pre-exposed to prolonged cocaine self-administration was not surmountable by increasing doses of cocaine (Fig. 5b, insert). Finally, the preference for lever S was so strong that it also emerged in rats under the influence of cocaine during choice (*N* = 10). In this experiment, rats had continuous access to lever C alone during 3 h per day. After acquisition of lever pressing (>20 responses/session), they were tested on a modified discrete-choice procedure which consisted of a continuous access to lever C alone for 1 hour, followed by 8 discrete choice trials under the S+/C+ condition. Though rats responded each day on lever C to self-administer cocaine during the hour preceding choice (Fig. 5c), they nevertheless rapidly acquired a robust preference for lever S (Fig. 5d). As shown in 3 representative individuals, there was an abrupt, within-session shift in behavior from lever C to lever S during choice (Fig. 5e).

## Discussion

Virtually all rats preferred saccharin over intravenous cocaine, a highly addictive drug. The preference for saccharin is not attributable to its unnatural ability to induce sweetness without subsequent caloric input because the same preference was also observed with an equipotent concentration of sucrose, a natural sugar. Importantly, the preference for saccharin sweet taste was not surmountable by increasing doses of cocaine and was observed despite either cocaine intoxication, sensitization or intake escalation – the latter being a hallmark of drug addiction [22], [34]. In addition, in several cases, the preference for saccharin emerged in rats which had originally developed a strong preference for the cocaine-rewarded lever. Such reversals of preference clearly show that in our setting, animals are not stuck with their initial preferences and can change them according to new reward contingencies. Finally, the preference for saccharin was maintained in the face of increasing reward price or cost, suggesting that rats did not only prefer saccharin over cocaine (‘liking’) but they were also more willing to work for it than for cocaine (‘wanting’). As a whole, these findings extend previous research [31], [32] by showing that an intense sensation of sweetness surpasses maximal cocaine stimulation, even in drug-sensitized and -addicted users. The absolute preference for taste sweetness may lead to a re-ordering in the hierarchy of potentially addictive stimuli, with sweetened diets (i.e., containing natural sugars or artificial sweeteners) taking precedence over cocaine and possibly other drugs of abuse.

Though very pronounced, the preference for saccharin in the S+/C+ condition was not exclusive. On average, rats selected lever C on about 15.6% of occasions (range between experiments: 7 to 23%) which, together with sampling doses, represent a total of 3 intravenous cocaine doses per day. This daily amount of cocaine self-administration is very low compared to what rats will spontaneously self-administer during the same period of time (i.e., about 30 doses). Interestingly, this very low amount of cocaine intake was nevertheless sufficient in itself to induce a rapid and strong drug sensitization (see below). In fact, even in the S+/C- condition, rats occasionally responded on lever C (8.3% of the time) which was not rewarded by cocaine in this condition. This residual level of responding on lever C is not surprising and is predicted by the matching law which refers to the well-documented tendency of animals or humans to distribute their behavior in proportion to the reward value of available options [39]. This interpretation suggests that even in the S+/C- condition, responding on lever C has some, though relatively weak, reward value. In the present study, the reward value of lever C in the S+/C- condition probably results from some partial stimulus generalization between lever S and lever C while, in the S+/C+ condition, it probably largely results from cocaine itself. Regardless of this residual tendency to choose lever C, the present study nevertheless clearly demonstrates that rats largely prefer lever S when it is rewarded by taste sweetness.

At first glance, the discovery that intense sweetness surpasses intravenous cocaine is difficult to conciliate with previous empirical and theoretical research on cocaine addiction. First, our findings seem to run counter to seminal research in monkeys showing that the large majority of individuals prefer high doses of intravenous cocaine over dry food, regardless of the amount of food available [40], [41] and even despite severe weight loss [42]. However, in most previous studies, except one [43], the food option contained no or only modest concentrations of sweet tastants, which probably explains why it was neglected in favor of high doses of cocaine. In addition, in those studies that employed lightly sweetened food pellets [41], the amount of effort required to obtain the food option was ten times higher than to obtain cocaine, thereby favoring drug choices. However, in one choice study, all monkeys clearly preferred, ceteris paribus, the highest dose of cocaine over a 1-g sucrose pellet [43]. The discrepancy between this latter study and the present study may suggest either that sweetened beverages are more rewarding than sweetened dry-foods (which may induce thirst in addition to reward) and/or that one 1-g sucrose pellet is not enough to surmount the rewarding effects of the highest doses of cocaine. Finally, one cannot rule out the possibility that this discrepancy could also reflect an inter-specific gap between rodents and primates, the latter being hypothetically more susceptible to cocaine reward than the former. Future research is needed to tease apart these different hypotheses. Nevertheless, the present study clearly demonstrates in rats–an animal species that readily self-administer cocaine and that develops most of the signs of addiction following extended drug access [34]–[36]–that the reward value of cocaine is bounded and does not surpass taste sweetness–a sensory-driven reward.

**Figure 6:**
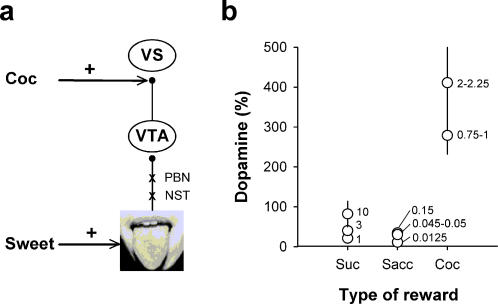
Effects of sucrose, saccharin or cocaine consumption on ventral striatal dopamine levels. a, Consumption of sweet solutions turns on midbrain dopamine cells that projects to the ventral striatum, possibly through a short, two-relay circuit in the brain stem [80]. In contrast, cocaine directly increases dopamine levels in the ventral striatum by blocking dopamine uptake. The symbol+indicates pharmacological or sensory stimulation and the symbol x, intermediate synapses. NST, nucleus of the solitary tract; PBN, parabrachial nucleus; VS, ventral striatum; VTA, ventral tegmental area. b, Mean (±SEM) levels of extra-cellular dopamine in the ventral striatum (expressed as percent change from baseline) during sucrose, saccharin or cocaine intake. These results are based on a meta-analysis of the literature (see Materials and Methods). Values that appear on the right of symbols represent sucrose or saccharin concentrations (in %) and cocaine doses (in mg/kg).

Our findings are also difficult to predict from current theorizing about the neurobiology of cocaine addiction. Despite considerable divergences, most influential theories of cocaine addiction (including recent neurocomputational models [44], [45]) postulate that cocaine is initially addictive through its direct and supranormal stimulation of dopamine signaling in the ventral striatum [15], [22], [46]–[49]. The repetition of this supranormal activation with repeated cocaine use would further increase the value of cocaine above that of other rewards, regardless of their initial value, thereby biasing decision-making towards excessive cocaine choice. This prediction is apparently contradicted by the present study. A meta-analysis of the literature (see Material and Methods) showed that intravenous cocaine self-administration was much more potent than sucrose or saccharin consumption in inducing dopamine levels in the ventral striatum in rats (Fig. 6). Despite its much greater neurochemical potency, however, we found that cocaine reward paled in comparison to sweet reward. In addition, the preference for saccharin developed despite a rapid and strong sensitization to the stimulant effects of cocaine–a well-documented behavioral phenomenon that is associated with long-lasting changes in striatal dopamine signaling [46], [47]. Thus, the ability of cocaine to directly boost midbrain dopamine neurons and to sensitize them durably is apparently not sufficient to make cocaine irresistible. This conclusion may somehow lead to a revision of some of the basic assumptions that underlie current neurobiological models of cocaine addiction.

First, our study may suggest that though much less efficacious in inducing presynaptic dopamine levels in the ventral striatum, sweet consumption may nevertheless generate an overall postsynaptic dopamine signal more intense than cocaine. The postsynaptic effects of supranormal levels of dopamine induced by cocaine are indeed probably limited by short-term receptor desensitization and/or inter- or intracellular opponent processes [15], [22]. Thus, absolute levels of striatal dopamine in response to different types of reward may not accurately predict their addictive potential. More direct measures of postsynaptic dopamine signaling will be required in the future to test this hypothesis. Alternatively, the absolute preference for intense sweetness may also point to the existence of brain signaling pathways that are more powerful than the mesostriatal dopamine pathway in controlling reward-oriented behavior and that taste sweetness would activate more vigorously than cocaine. Striatal opioid peptides are currently the best candidates to perform this function. Striatal gene expression of opioid peptides is modulated by overconsumption of sweetened water [50], [51] and pharmacological activation of ventral striatal opioid receptors, especially of mu receptors, increases the intake and palatability of sweetened water [52], [53]. What is less clear at present, however, is whether activation of striatal opioid signaling can override dopamine signaling in the control of behavior. One way to address this question would be to allow rats to choose between cocaine and a drug manipulation that selectively boosts striatal opioid signaling. A more general approach would be to use brain imaging technologies to search for regions or networks that respond more to taste sweetness than to intravenous cocaine. Finally, it is also possible that taste sweetness surpasses cocaine simply because the latter has more negative side-effects and thus is more conflictual or ambivalent than the former [54]. Indeed, besides activating striatal dopamine signaling, cocaine also activates brain stress pathways, such as the extra-hypothalamic corticotropin-releasing factor pathways which play a critical role in fear and anxiety [55]. The concurrent activation of brain stress pathways by cocaine could explain why initially drug-naïve rats were more hesitant in sampling the cocaine-rewarded lever than the saccharin-rewarded lever in the present study. In addition, the ambivalent effects of cocaine may also contribute to explain why rats in the S+/C+ condition developed a reliable preference for lever S more rapidly that rats in the S+/C- condition (day 2 *versus* day 7). However, this ambivalence hypothesis is unlikely to explain the preference for taste sweetness in cocaine-escalated rats which did no longer show this hesitation (latency to sample lever C: 15.6±8.1 s; latency to sample lever S: 4.0±0.8 s; *F*(1,10) = 2.06, NS), presumably because of a tolerance to the stressful or anxiogenic effects of cocaine.

Whatever the mechanisms involved, the discovery that intense sweetness takes precedence over cocaine, one of the most addictive and harmful substance currently known [33], suggests that highly sweetened beverages, such as those widely available in modern human societies, may function as supernormal stimuli [56]. By definition, a supernormal stimulus is more effective than naturally occurring stimuli in controlling behavior and therefore can override normal behaviors (e.g., host-bird parents succumbing to the supernormal begging call of an insatiable nestling cuckoo to the detriment of their own offspring [57]). Sweet taste perception depends on two G-protein-coupled subunit receptors, T1R2 and T1R3 [1], [2]. In most mammals, including rodents and primates, these receptors have evolved in ancestral environments poor in sugars and are thus not adapted to high concentrations of sweet tastants [1], [2]. We speculate that the supranormal stimulation of these receptors by highly-sweetened diets generates a supranormal reward, with the potential to override both homeostatic and self-control mechanisms and thus to lead to addiction [58]. Finally, the present study may also suggest that the current, widespread availability of sugar-rich diets in modern human societies may provide an unsuspected, though highly costly, shield against the further spread of drug addiction. Future research on animals reared in sugar-enriched environments, to better approximate the modern human condition, may provide important clues to address this important issue.

## Materials and Methods

### Subjects

Naïve, young adult (221–276 g), male, Wistar rats (*N* = 132) were used in the present study (Charles River, France). Rats were housed in groups of two or three and were maintained in a light- (12-h reverse light-dark cycle) and temperature-controlled vivarium (22°C). All behavioral testing occurred during the dark phase of the light-dark cycle. Food and water were freely available in the home cages. Food consisted of standard rat chow A04 (SAFE, Scientific Animal Food and Engineering, Augy, France) that contained 60% of carbohydrates (largely corn starch), 16% of proteins, 12% of water, 5% of minerals, 3% of fat and 4% of cellulose. No synthetic or refined sugar was added. All experiments were carried out in accordance with institutional and international standards of care and use of laboratory animals [UK Animals (Scientific Procedures) Act, 1986; and associated guidelines; the European Communities Council Directive (86/609/EEC, 24 November 1986) and the French Directives concerning the use of laboratory animals (décret 87-848, 19 October 1987)].

### Apparatus

Twelve identical operant chambers (30×40×36 cm) were used for all behavioral training and testing (Imétronic, France). All chambers were located away from the colony room in a dimly lit room. They were individually enclosed in wooden cubicles equipped with a white noise speaker (45±6 dB) for sound-attenuation and an exhaust fan for ventilation. Each chamber had a stainless-steel grid floor that allowed waste collection in a removable tray containing maize sawdust. Each chamber was constituted of two opaque operant panels on the right and left sides, and two clear Plexiglas walls on the rear and front sides (the front side corresponds to the entry/exit of the chamber). Each operant panel contained an automatically-retractable lever, mounted on the midline and 7 cm above the grid. The left operant panel was also equipped with a retractable, cylinder-shaped drinking spout, 9.5 cm to the left of the lever and 6 cm above the grid. A lickometer circuit allowed monitoring and recording of licking. A white light diode (1.2 cm OD) was mounted 8.5 cm above each lever (from the center of the diode). Each chamber was also equipped with two syringe pumps placed outside, on the top of the cubicle. One syringe pump was controlled by the left lever and delivered water or saccharin (or sucrose) solution into the drinking spout through a silastic tubing (Dow Corning Corporation, Michigan, USA). The other pump was controlled by the right lever and delivered drug solution through a Tygon tubing (Cole Parmer) connected via a single-channel liquid swivel (Lomir biomedical inc., Quebec, Canada) to a cannula connector (Plastics One, Roanoke, VA) on the back of the animal. The Tygon tubing was protected by a stainless-steel spring (0.3 cm ID, 0.5 cm OD) (Aquitaine Ressort, France) which was suspended at the center of the chamber from the swivel tether connector. Vertical movements of the animal were compensated for by means of a counterbalancing weight-pulley device.

### Surgery

Anesthetized rats (Chloral hydrate, 500 mg/kg IP) (J-T Baker, The Netherlands) were prepared with silastic catheters (Dow Corning Corporation, Michigan, USA) in the right jugular vein that exited the skin in the middle of the back about 2 cm below the scapulae. After surgery, catheters were flushed daily with 0.15 ml of a sterile antibiotic solution containing heparinized saline (280 IU/ml) (Sanofi-Synthelabo, France) and ampicilline (Panpharma, France). When needed, the patency of the catheter was checked by administering 0.15 ml of the short-acting non-barbiturate anesthetic etomidate through the catheter (Braun Medical, France). Behavioral testing began 7–10 days after surgery.

### Discrete-trials choice procedure

Each day, rats were allowed to choose between a cocaine-paired lever (lever C) and a saccharin-paired lever (lever S) on a discrete-trials choice procedure. Cocaine reward consisted of one i.v. dose of 0.25 mg delivered over 4 s. This dose is widely used in rats and was used in all of our previous self-administration studies [34], [35]. Saccharin reward consisted of a 20-s access to a drinking spout that delivered discrete volumes (0.02 ml) of a solution of sodium saccharin at a near optimal concentration of 0.2% [59], [60]. The first 3 volumes were delivered freely during the first 3 s to fill the drinking spout; subsequent volumes were obtained by licking (1 volume per 10 licks in about 1.4 s). Thus, during a 20-s access to saccharin solution, a maximum of 15 volumes could be obtained which corresponds to 0.3 ml. Rats learned to drink this maximum amount per access within the first week of testing.

Each choice session was constituted of 12 discrete trials, spaced by 10 min, and divided into two successive phases, sampling (4 trials) and choice (8 trials). During sampling, each trial began with the presentation of one single lever in this alternative order: C–S–C–S. Lever C was presented first to prevent an eventual drug-induced taste aversion conditioning or negative affective contrast effects. If rats responded within 5 min on the available lever, they were rewarded by the corresponding reward. Reward delivery was signaled by retraction of the lever and a 40-s illumination of the cue-light above this lever. If rats failed to respond within 5 min, the lever retracted and no cue-light or reward was delivered. Thus, during sampling, rats were allowed to separately associate each lever with its corresponding reward (lever C with cocaine, lever S with saccharin) before making their choice. During choice, each trial began with the simultaneous presentation of both levers S and C. Rats had to select one of the two levers. During choice, reward delivery was signaled by retraction of both levers and a 40-s illumination of the cue-light above the selected lever. If rats failed to respond on either lever within 5 min, both levers retracted and no cue-light or reward was delivered.

### Acquisition of lever preference

To assess the acquisition of a preference for either lever, operant naïve, non-restricted animals were tested during 15 consecutive days under the 3 reward conditions described in the main text (one group of rats per condition). Under each reward condition, the response requirement of each reward was initially set to 1 response (first 10 days) and then incremented to 2 consecutive responses to avoid eventual accidental choice (remaining days). When the response requirement was 2, a response on either lever reset the response requirement on the other lever. Response resetting occurred very rarely, however.

### Effects of cocaine on locomotion

Each self-administration chamber was also equipped with two pairs of infrared beams 2 cm above the grid floor (Imétronic, France). Both pairs crossed the chamber on its length axis and were separated from each other by 16 cm, and from the right or left wall by 12 cm. This placement allowed one to count the number of horizontal displacements of the animal to go to and fro between the two extremities of the length axis (cage crossings).

### Effects of cocaine doses on choice

After behavior stabilization under the S+/C+ condition (no increasing or decreasing trends over 3 consecutive days), a subgroup of rats (*N* = 11) were tested with increasing i.v. doses of cocaine (0.25, 0.75 and 1.5 mg). Each dose was obtained by increasing the drug concentration and was delivered intravenously over 4 s. During continuous cocaine self-administration, the spontaneous inter-injection interval–which reflects the duration of cocaine effects–increases non-linearly with the unit dose available. In our conditions, the inter-injection interval was on average 4.3, 10.7 and 17.4 min for 0.25, 0.75 and 1.5 mg, respectively [61]. Thus, to maintain the same conditions of choice across doses (i.e., same delay between end of drug effects and next choice) and to avoid drug accumulation, the inter-trial interval was increased with the dose: 10 (4.3+5.7), 16.4 (10.7+5.7) and 23.1 (17.4+5.7) min for 0.25, 0.75 and 1.5 mg, respectively. Each dose was in effect for at least 5 consecutive days. Average behavior at each dose was considered stable when there was no increasing or decreasing trends over 3 consecutive days.

### Estimation of delay of onset of cocaine effects

Though the intravenous route of administration allows for rapid drug action, there is nevertheless a short and incompressible delay between the response and the onset of drug effects. This delay was estimated here by timing the first observable behavioral reaction to cocaine following the onset of drug delivery. Each rat responds to i.v. cocaine in a very characteristic fashion: it frantically runs around the cage while brushing rapidly its vibrissae with its forepaws, the head and neck lowered to the floor (Ahmed, unpublished observations). This observation was conducted in a subgroup of rats (*N* = 12) before and after testing under the S+/C+ condition. On both occasions, the mean delay of onset of cocaine effects was 6.2±0.2 s.

### Effects of delay of saccharin reward on choice

After behavior stabilization under the S+/C+ condition (no increasing or decreasing trends over 3 consecutive days), a subgroup of rats (*N* = 11) were tested with increasing delays between behavior and saccharin delivery (0, 6, 12 and 18 s). The 6-s delay corresponds to the delay of onset of cocaine effects, as measured through direct observation (see below). Each delay was in effect for at least 5 consecutive days. Average behavior at each delay was considered stable when there was no increasing or decreasing trends over 3 consecutive days.

### Effects of reward price on choice

After behavior stabilization under the S+/C+ condition (no increasing or decreasing trends over 3 consecutive days), a subgroup of rats (*N* = 10) were tested with increasing reward prices or response requirements (2, 4 and 8 consecutive responses). Each response requirement was tested for at least 5 consecutive days. At each requirement, a response on either lever reset the response requirement on the other lever. Average behavior at each price was considered stable when there was no increasing or decreasing trends over 3 consecutive days.

### Induction of cocaine intake escalation

Rats (*N* = 11) had prolonged access to cocaine self-administration (i.e., 6 h per day during 18 days) before being allowed to choose between cocaine and saccharin. Daily access to cocaine was contingent on a fixed-ratio time-out 40s schedule, that is a fixed number of responses (see below) was required to earn a unit dose with a minimum inter-dose interval of 40s. The unit dose of cocaine was 0.25 mg during the first hour and 0.75 mg during the last 5 hours. The increase of the unit dose of cocaine during the last 5 hours was intended to speed up and to aggravate cocaine intake escalation. The response requirement was initially set at 1 response/dose (first 14 days) and then incremented to 2 responses/dose (remaining days). The day after cocaine intake escalation, rats were allowed to choose between cocaine and saccharin during 10 consecutive days on the discrete-trials choice procedure described above (S+/C+ condition).

### Choice during cocaine intoxication

Rats (*N* = 10) were first trained to self-administer cocaine 3 hours per day during 1 week, under a fixed-ratio schedule of reinforcement, with a time-out of 40 s. The response requirement was initially set at 1 response/dose (first 3 days) and then incremented to 2 responses/dose (remaining days). Then, rats were tested under a modified discrete-trials choice procedure. The sampling period of the original procedure was replaced by a 1-h continuous access to lever C alone during which rats could obtain cocaine according to a fixed-ratio 2 time-out 40 s schedule. Except that, the novel procedure was identical to the original (described in the main text). Thus, each day, rats were under the influence of cocaine (i.e., cocaine-intoxicated) before making their 8 choices between lever S and lever C (S+/C+ condition).

### Meta-analysis: effects of sucrose, saccharin or cocaine consumption on striatal dopamine levels

A Medline search was conducted, using the following keywords: rat, cocaine, saccharin, sucrose, self-administration, dopamine, microdialysis, striatum, accumbens. Retrieved articles were checked and sorted out according to content and relevance. At the end, a total of 18 papers [62]–[79] were kept for graphical analysis. In each case, the effects of sucrose, saccharin or cocaine consumption on extracellular dopamine levels in the ventral striatum were estimated from the figures.

### Drugs

Cocaine hydrochloride (Coopération Pharmaceutique Française, France) was dissolved in 250-ml or 500-ml sterile bags of 0.9% NaCl and kept at room temperature (21±2°C). Drug doses were expressed as the weight of the salt. Sodium saccharin (Sigma-Aldrich, France) was dissolved in tap water at room temperature (21±2°C). The saccharin's solution was renewed each day.

### Data analysis

For convenience, the indifference level between lever S and lever C was set at 0. Values above 0 indicated a preference for lever S (i.e., selection of lever S>50% of completed choice trials) while values below 0 indicated a preference for lever C (i.e., selection of lever C>50% of completed choice trials). Some rats had to be excluded from the study because they failed to acquire the operant behavior (i.e., 20 out 132 rats whose 16 in the S-/C+ condition and 4 in the S+/C+ condition). Specifically, these rats completed less than 50% of the 8 daily choice trials after 15 days of testing, a choice performance too low to allow a reliable measurement of their preferences. Statistical analyses were run using Statistica, version 7.1 (Statsoft, Inc France).
